# Time delays in treatment of snakebite patients in rural Sri Lanka and the need for rapid diagnostic tests

**DOI:** 10.1371/journal.pntd.0008914

**Published:** 2020-11-30

**Authors:** Anjana Silva, Jiri Hlusicka, Nipuna Siribaddana, Subodha Waiddyanatha, Senaka Pilapitiya, Prasanna Weerawansa, Niroshan Lokunarangoda, Sujeewa Thalgaspitiya, Sisira Siribaddana, Geoffrey K. Isbister

**Affiliations:** 1 Faculty of Medicine and Allied Sciences, Rajarata University of Sri Lanka, Saliyapura, Sri Lanka; 2 Monash Venom Group, Faculty of Medicine, Nursing and Health Sciences, Monash University, Australia; 3 South Asian Clinical Toxicology Research Collaboration, University of Peradeniya, Peradeniya, Sri Lanka; 4 Charles University in Prague, 1st Faculty of Medicine, 4th Department of Internal Medicine, Prague, Czech Republic; 5 Clinical Toxicology Research Group, School of Medicine and Public Health, University of Newcastle, Newcastle, New South Wales, Australia; College of Health Sciences, Bayero University Kano, NIGERIA

## Abstract

Delays in treatment seeking and antivenom administration remain problematic for snake envenoming. We aimed to describe the treatment seeking pattern and delays in admission to hospital and administration of antivenom in a cohort of authenticated snakebite patients. Adults (> 16 years), who presented with a confirmed snakebite from August 2013 to October 2014 were recruited from Anuradhapura Hospital. Demographic data, information on the circumstances of the bite, first aid, health-seeking behaviour, hospital admission, clinical features, outcomes and antivenom treatment were documented prospectively. There were 742 snakebite patients [median age: 40 years (IQR:27–51; males: 476 (64%)]. One hundred and five (14%) patients intentionally delayed treatment by a median of 45min (IQR:20-120min). Antivenom was administered a median of 230min (IQR:180–360min) post-bite, which didn’t differ between directly admitted and transferred patients; 21 (8%) receiving antivenom within 2h and 141 (55%) within 4h of the bite. However, transferred patients received antivenom sooner after admission to Anuradhapura hospital than those directly admitted (60min [IQR:30-120min] versus 120min [IQR:52-265min; p<0.0001]). A significantly greater proportion of transferred patients had features of systemic envenoming on admission compared to those directly admitted (166/212 [78%] versus 5/43 [12%]; p<0.0001), and had positive clotting tests on admission (123/212 [58%] versus 10/43 [23%]; p<0.0001). Sri Lankan snakebite patients present early to hospital, but there remains a delay until antivenom administration. This delay reflects a delay in the appearance of observable or measurable features of envenoming and a lack of reliable early diagnostic tests. Improved early antivenom treatment will require reliable, rapid diagnostics for systemic envenoming.

## Author summary

Snakebite is a neglected tropical disease which is closely associated with underdevelopment. Poor accessibility to safe and effective antivenoms is a major issue in some regions highest-affected by snakebites. Antivenom can prevent severe effects of envenoming if given early. Therefore, educating communities and health care workers to improve treatment seeking after snakebite is a global priority. Many factors are associated with delayed presentation to hospital and early administration of antivenom. We found that most snakebite patients in Sri Lanka present to the first hospital within an hour of the bite. However, there remains a delay of about two more hours until the first dose of antivenom is administered. This delay is a reflection of a delay in the appearance of observable or measurable features of envenoming and a lack of reliable early diagnostic tests. We emphasise the need for reliable, rapid diagnostics for systemic envenoming.

## Introduction

Snakebite remains an important public health problem in the tropics, disproportionately affecting poor rural populations working primarily in regions where there is a high human-snake interaction[1]. The World Health Organisation (WHO) declared snakebite as a neglected tropical disease in 2017 [2]. The true global burden of snake envenoming is unknown, but literature-based estimates suggest that about five million snakebites and roughly two million envenomings occur annually worldwide[3]. In addition to the life-threatening acute effects, the long-term effects suffered by the survivors also create a socioeconomic burden in the affected regions [4,5]. Antivenom is the only specific treatment available for snakebite globally [6].

High quality data is required to address issues in the early treatment of envenoming, including the supply of antivenom and its safety[5]. Many of these issues are specific to different regions and communities, due to the uniqueness of the endemic snake species, and varying human behaviours that predispose to snakebite [7,8]. Understanding the local socio-demographic factors leading to snake envenoming is important in planning snakebite mitigation activities [9]. Inaccessibility to healthcare facilities, seeking alternative treatments and home remedies can delay the presentation of snakebite patients.

The evidence for the association between delayed presentation and increased severity of envenoming has been demonstrated many times, with shorter times to antivenom being associated with improved patient outcomes [10,11]. There are two major delays in the administration of antivenom. The first is any delay from the bite to arrival in a hospital in which antivenom can be administered. The second is any delay between systemic envenoming (venom reaching the systemic circulation) and recognition of systemic envenoming clinically (symptoms or signs) or on abnormal blood tests. Encouraging early presentation to primary health care centres has been key to improving snakebite outcomes globally[12]. Despite this, delays in presentation to a healthcare setting continue to be a major problem in regions such as South Asia, with only 50% of patients presenting to hospitals within six hours of the bite[9]. More problematic is improving the early diagnosis of systemic envenoming so that antivenom can be administered as soon as possible to patients in hospital.

Sri Lanka has one of the highest snakebite incidences in the world[13]. Unlike other parts of South Asia, people usually seek standard medical care first and are more aware of the appropriate first aid therapies [14]. The time to arrival in hospital has also been reduced over the last three decades, decreasing the mortality and proportion of patients with complications[10]. However, despite patients presenting early, there remains issues with the early diagnosis of envenoming [15] and lack of geographically specific, low-reactogenic antivenom for Sri Lankan snakes[16]. Concerns about antivenom reactions contributes to delays in antivenom administration in patients who initially have no or minimal evidence of systemic envenoming.

We aimed to describe the treatment seeking pattern of snakebite patients and the time to both hospital presentation and antivenom administration in a cohort of authenticated snakebite patients admitted to a tertiary care centre in Sri Lanka.

## Methods

### Ethics statement

Ethics approval for the cohort study was obtained from the Ethics Review Committee of the Rajarata University of Sri Lanka (04/09/2013) and the Monash University Human Research Ethics Committee (CF14/970–2014000404). Written informed consent was obtained from all patients.

### Study design and setting

We undertook a retrospective review of a prospective cohort of patients presenting with snakebites to the Teaching Hospital Anuradhapura. This is the third largest medical facility in the country. Anuradhapura hospital covers the North Central Province, which is a large geographical area and records the highest incidence of snake envenoming in the country.

### Patients

Patients aged 16 years or greater, who presented between August 2013 and October 2014 with a confirmed snakebite to Anuradhapura hospital were recruited. The bite was confirmed if the patient had identifiable fang/teeth marks, features of local or systemic envenoming or the patient witnessed the snake bite. If the snake specimen was available, it was identified by an experienced herpetologist (AS). In the remaining patients, species specific sandwich enzyme-linked immunosorbent assay (ELISA) was conducted on pre-antivenom blood samples to determine the snake species and the data was previously reported [10,17]. In cases in which a pre-antivenom blood sample was not available, ELISA was done on the sample following venom dissociation as previously described[18].

### Treatment

All patients were managed by the treating physicians at the study hospital. Administration of Indian polyvalent antivenom (Bharat serums and vaccines limited and Vins Bioproducts) was determined by the treating physicians based on the evidence of systemic envenoming (neurotoxicity, coagulopathy) and/or severe local envenoming, as per the guidelines of the Sri Lankan Medical Association[19]. Patients requiring intubation and mechanical ventilation were admitted to the intensive care unit.

### Data collection

All patients recruited to the snakebite cohort have data prospectively collected including demographics, information on the circumstances of the bite, prehospital information (first aid, health seeking behaviour, previous admissions to local hospitals), clinical features, laboratory investigations, outcomes and treatment, including antivenom therapy. The information is collected on a clinical research form (S1 Data) during the hospital stay by clinical research assistants. This is then entered into a relational database. Data were then extracted for this retrospective review.

### Statistical analysis

Continuous data were described using median and interquartile ranges (IQR) and were analysed using non-parametric statistical methods in GraphPad PRISM 8.3 (GraphPad Software, San Diego, CA, USA). Proportions were compared with Fisher’s exact test and continuous outcomes with the Mann-Whitney test. Hierarchical multiple regression was used to investigate the association between several variables (“patient eye-witnessing the bite”, “under the influence of alcohol when bitten”, “admitted directly to the Anuradhapura hospital or admitted to the peripheral hospital”, “snake bought to the hospital”) and the time from bite to any hospital admission, after controlling for occupation, sex, education and age. Preliminary analysis was conducted to ensure no violation of the assumptions of normality, linearity, multicollinearity, and homoscedasticity using IBM SPSS Statistics for Windows, Version 21.0. Armonk, NY.

## Results

**Fig 1:**
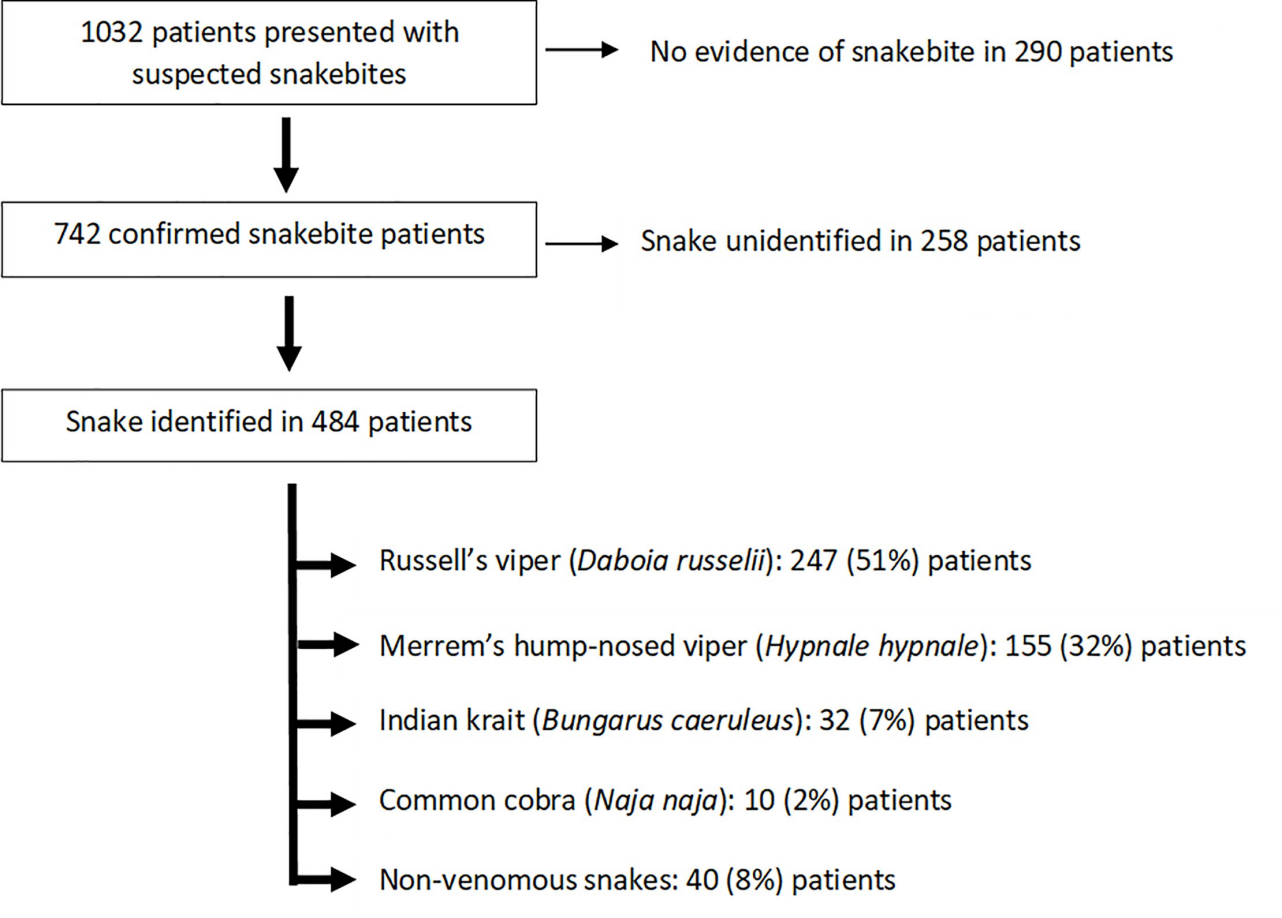
Flow diagram showing the enrolment of patients for the present study.

During the study period, there were 1032 patients that presented with suspected snake bites. Of these, 742 were confirmed snakebites, and were included in this study analysis [median age: 40 years (IQR 27 - 51y; males: 476 (64%); Fig 1]. The species of snake was determined in 484 (65%) patients. There were 554 patients (75%) who were transferred from a primary or secondary care hospital to Anuradhapura hospital, while the remaining 188 (25%) were direct admissions to the study hospital. The clinical effects and outcomes for the patients are included in the Table 1.

**Table 1:** The clinical effects and outcomes of the 742 snakebite patients recruited for the study.

	Identified n = 485					Unidentified n = 257
	Russell’s viper n = 247	Hump-nosed viper n = 155	Indian Krait n = 33	Indian Cobra n = 10	Non-venomous n = 40	
Length of Stay (median + IQR)	3 (2–4)	1 (1–3)	5 (3–8)	3 (1–4)	1	3 (2–4)
Local envenoming: n, (%)	244 (99)	148 (95)	-	7 (70)	-	123 (48)
VICC: n, (%)	166 (67)	8 (5)	-	-	-	81 (32)
Neurotoxicity: n, (%)	130 (53)	-	25 (76)	1 (10)	-	32 (12)
Mechanical ventilation: n, (%)	5 (2)	-	17 (52)	-	-	2
AKI requiring dialysis: n, (%)	5 (2)	1	-	-	-	1
Deaths: n, (%)	6 (2)	-	-	-	-	1
Left against medical advice: n, (%)	1	2	-	-	2 (5)	7 (3)
Amputations: n, (%)	-	1	-	-	-	-

VICC–venom induced consumption coagulopathy; AKI–acute kidney injury

### Treatment seeking

A total of 105 (14%) patients intentionally delayed seeking hospital treatment (Table 2), while the remainder presented to a local primary hospital or the study hospital directly. The median time to hospital presentation for those intentionally delaying presentation was 45 min (IQR: 20–120 min) and the commonest cause for the intentional delay was waiting for the onset of symptoms (Table 2). Patients with venomous snakebites only intentionally delayed treatment seeking for a median of 20 min (IQR: 10–90 min) compared to those with non-venomous and unidentified snakebites, who delayed treatment by 45 min (IQR: 30–120 min).

**Table 2:** Reasons for presentation delays and the time spent for the delays of presentation in in 105 patients who intentionally delayed treatment seeking.

Reasons for Delayed Treatment Seeking	Number of patients (%)	Time spent in minutes: median (IQR)
Waiting for appearance of symptoms	52 (50)	120 (30–360)
Seeking native treatment	15 (14)	70 (20–345)
Catching the snake	24 (23)	10 (10–20)
Waiting for someone to accompany them to the hospital	14 (13)	60 (40–90)

### Time from bite to admission

**Fig 2:**
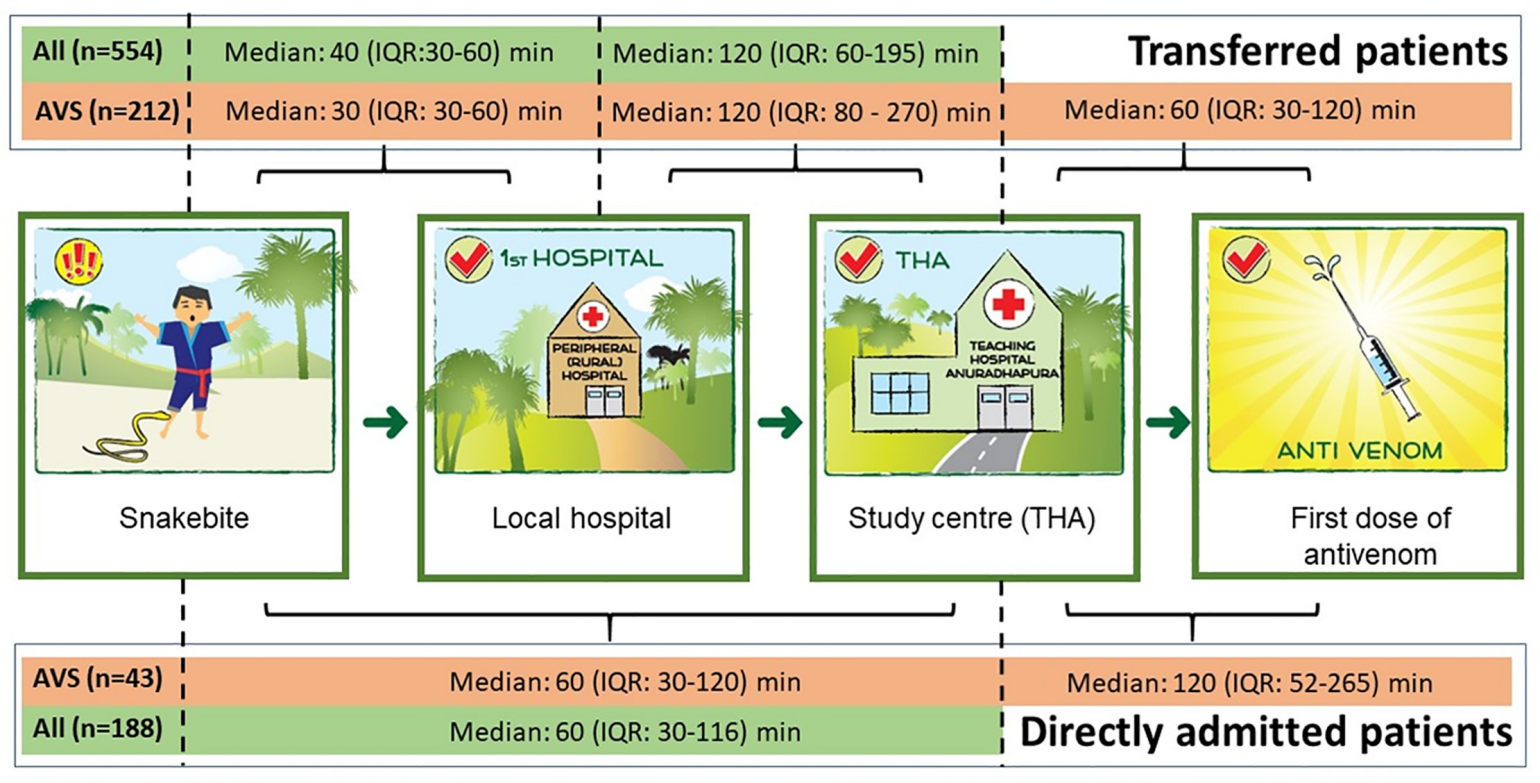
Median times from the bite until the first dose of antivenom at the study hospital, including the different time gaps for transferred patients (n = 534) and directly admitted patients (n = 188).

The median time to presentation for patients presenting directly to Anuradhapura hospital was 60 min (IQR: 30–116 min). This was longer than those presenting directly to a local hospital with a median time to presentation of 40 min (IQR 30–60). However, those initially presenting peripherally ultimately presented later to Anuradhapura hospital at a median time of 150 min (IQR: 90–195 min), after interhospital transfer (Fig 2).

A hierarchical multiple regression model was developed to assess the factors that were associated with the time from bite to any hospital admission. The model included occupation, sex, education and age, which explained 0.5% of the variance. After inclusion of the variables–“patient eye-witnessing the bite”, “under the influence of alcohol when bitten”, “admitted directly to the Anuradhapura hospital or admitted to the peripheral hospital”, “snake bought to the hospital”—the total variance explained by the model increased to 3.9%, (*F* (8,663) = 3.36 *p* < 0.001). In the final model, only *patient eye-witnessed the bite* was statistically significant (S1 and S2 Tables).

### Time to antivenom

Two hundred and fifty-five patients (34%) received Indian polyvalent antivenom. The first dose of antivenom was administered a median of 230 min (IQR 180–360 min) post-bite at the study hospital. The time from bite to first dose of antivenom did not differ between directly admitted patients (median 230min; IQR: 120–410 min) and transferred patients (median 220 min; IQR: 180–290 min; p>0.05, Mann-Whitney test). Twenty-one patients (8%) received the first dose of antivenom within two hours and 141 (55%) received the first dose within four hours of the bite. However, transferred patients received antivenom sooner after admission to Anuradhapura hospital than those directly admitted, with a median time from admission to antivenom of 60 min (IQR:30–120) compared to 120 min (IQR: 52–265 min; p<0.0001, Mann-Whitney test).

Of the 255 patients receiving antivenom, 171 (67%) received antivenom based on the presence of bleeding manifestations (n = 45, 18%), neurological manifestations (n = 138, 54%) or positive bed-side clotting tests (n = 128, 50%) on admission. The remainder (n = 84, 33%) received antivenom based on a subsequently positive bed-side clotting test or development of clinical features of systemic envenoming. Of those who received antivenom, a significantly larger proportion of transferred patients had clinical features of systemic envenoming on admission compared to those directly admitted (166/212 [78%] versus 5/43 [12%]; absolute difference: 67%; 95% confidence intervals [95% CI]: 58–82%; p<0.0001). Further, of those who received antivenom, a significantly greater proportion of transferred patients had clotting tests positive on admission, compared to those directly admitted (123/212 [58%] versus 10/43 [23%]; absolute difference: 34%; 95% CI: 22–52%; p<0.0001). Ninety patients (35%) developed acute adverse reactions to antivenom.

## Discussion

Our study showed that in rural Sri Lanka, patients present early to hospital after a snakebite and more than half received antivenom within 4 hours. Interestingly, this is the same for patients presenting directly to a major hospital and for those who present to a peripheral hospital and are then transferred to receive antivenom. There were only a small proportion of patients who intentionally delayed seeking treatment. Although not specifically investigated in the study, there appeared to be a delay in the onset of clinical effects or abnormal laboratory tests indicative of envenoming. Patients who were transferred, and therefore who arrived much later to the study hospital 2.5 hours post-bite, compared to 1 hour, were significantly more likely to have evidence of systemic envenoming. Consistent with this, these transferred patients received antivenom sooner after admission, compared to directly admitted patients.

In over three quarters of transferred patients, clinical features of systemic envenoming had already manifested on admission to the study hospital. This is the reason that these patients got antivenom soon after they arrived at the study hospital. In contrast, only a small fraction of directly admitted patients had clinical features of systemic envenoming on admission. Therefore, despite reaching the study hospital in a median time of one hour from the bite, the directly admitted patients had to wait twice as long to receive antivenom because of a delay in the development of clinical features of envenoming. This suggests that the most plausible reason for the delay in antivenom administration, in spite of early presentation of the patients, is the delay in diagnosis. Medical staff has to wait for the appearance of specific clinical manifestations of envenoming. Although manifestation of the features of non-specific systemic envenoming have been shown to be early predictors of systemic envenoming [20], such features are not use in deciding antivenom therapy in Sri Lanka as yet[19].

A substantial proportion of patients developed acute adverse reactions to antivenom in this cohort. Acute adverse reactions to Indian polyvalent antivenom are a major problem in Sri Lanka [16], and because of the plethora of data and subsequent strain on health service professionals in managing the adverse reactions, doctors are hesitant to administer antivenom until they have absolute certainty in their diagnosis. This contributes to the delay in antivenom administration, with doctors waiting for concrete evidence of envenoming before they administer antivenom.

We have shown that an average patient who presents to a hospital in Sri Lanka after a snakebite spends more time within the healthcare system waiting to receive antivenom, than the time from the bite to hospital admission. However, it appears that it may not actually be delays in patient treatment that cause this, but a delay in the onset of envenoming, or a delay in observable or measurable features of systemic envenoming. We found that there were a group of patients who present to hospital “too” early, in that they have no clinical symptoms or signs of envenoming or abnormal blood tests on arrival, and there is a delay in antivenom administration until they develop evidence of envenoming. This may simply be a reflection of a slower onset for viper envenoming due to deeper venom injection causing a depot effect–the slower and longer release of venom into the systemic circulation. We have shown previously that 60% of Russell’s viper bite patients from this cohort who did not have neurotoxicity on admission, developed neurotoxicity over the next eight hours[10]. Even so, early administration of antivenom can only occur if there is a way to detect the presence of venom in the systemic circulation, before there is clinical or laboratory evidence of venom effects.

The low mortality rate of patients in this cohort most likely reflects the early presentation of the patients to the healthcare system and rapid transportation of the patients from the local hospitals to the tertiary care centre, enabling early antivenom and supportive care. In fact, the median time to antivenom for all patients of 3.8 h was similar or slightly better than for Australian snakebite patients of 4.3 hours (IQR, 2.7 to 6.3 h)[21]. For most of the developing world, presentation of many snakebite victims to the healthcare facilities is delayed due to many socio-cultural reasons such as poor accessibility, lack of awareness, cultural beliefs and poverty [9,22]. The promotion of presentation to health care system early, following snakebites is included in the recent strategy for prevention and control of envenoming put forward by the World Health Organisation, under the objective of “empowering and engaging communities” [12,23].

Sri Lanka has a fairly well-developed public health system and a comparatively better social capital with better literacy, which has led to the elimination of tropical conditions such as malaria and lymphatic filariasis [24,25]. The at-risk population in Sri Lanka, have a better understanding and awareness of the principles and benefits of proper and rapid treatment seeking [14,26,27]. In the community-based studies it was shown that 70% of snakebite patients in Sri Lanka seek allopathic medicine and those who develop probable envenoming are much more likely to seek allopathic treatment [26]. In this cohort, the intentional delays of treatment seeking by the patients was seen only in 14% of the patients. The time loss for intentional delays in patients with venomous snake bites was shorter than the others. In particular, only 2% of the patients sought native treatment before admission to hospital, reflecting a further improvement in treatment seeking behaviour. This has been one of the major successes in the treatment of snakebite in Sri Lanka over the last few decades, confirmed by a snakebite mortality rate as low as 0.5%, despite having a highly reactogenic antivenom[13,16,26]. Although a positive finding for Sri Lanka, this unique situation in Sri Lanka of accessible healthcare may not be generalisable for many developing countries.

When the health seeking behaviours of communities are improved, as in case of this cohort, the diagnosis and treatment of envenoming must be developed in parallel. Therefore, reliable early diagnostic tests must be available for the patients to receive the benefit of early presentation to hospital. The lack of reliable, early diagnostics of envenoming and the delays of the diagnosis of envenoming is related to the reliability of the widely used cheap and simple WBCT20 [5,15]. The utility of early detection of systemic envenoming based on the detection of universal toxin groups, such as phospholipases A_2_ has been previously shown, but needs to be developed in an affordable, bedside test [28]. Hence, we call for rapid, standardized affordable diagnostic tests as a priority, as a part of the global research agenda of snake bite. However, we must acknowledge that the present study was not specifically designed to assess the effect of potential human and health system delays on the delay in antivenom therapy. We cannot simply assume that the delays in antivenom therapy are exclusively due to the diagnostic tests.

It appears in Sri Lankan snakebite that the onset of symptoms or abnormal bedside/laboratory tests takes longer than the median time to hospital presentation of 40 to 60 minutes. This demonstrates the effectiveness of public health measures over the last few decades in getting patients to seek hospital treatment early, rather than traditional medicines. However, it also shows that improvements need to be made in the early diagnosis of systemic envenoming. The development of bedside testing for the presence of venom in the circulation will be key to this.

## Supporting information

S1 Table Hierarchical regression model summary. (DOCX)

S2 Table Variables in the hierarchical regression model contributing to the bite to admission time (dependent variable). (DOCX)

S1 Data Snake bite data collection sheet.pdf. (PDF)
