# Supplementary material for: Time delays in treatment of snakebite patients in rural Sri Lanka and the need for rapid diagnostic tests
Source: PLoS Negl Trop Dis. 2020 Nov 30;14(11):e0008914. doi: 10.1371/journal.pntd.0008914 (PMC7728389; doi:10.1371/journal.pntd.0008914)
Supplement: S1 Table — (DOCX) [file pntd.0008914.s001.docx]

| **Model Summary^c^** | | | | | | | | | |
| --- | --- | --- | --- | --- | --- | --- | --- | --- | --- |
| Model | R | R Square | Adjusted R Square | Std. Error of the Estimate | Change Statistics | | | | |
|  |  |  |  |  | R Square Change | F Change | df1 | df2 | Sig. F Change |
| 1 | .069^a^ | .005 | -.001 | 263.235 | .005 | .787 | 4 | 667 | .534 |
| 2 | .197^b^ | .039 | .027 | 259.438 | .034 | 5.917 | 4 | 663 | .000 |
| a. Predictors: (Constant), occupation, sex, education, Age | | | | | | | | | |
| b. Predictors: (Constant), occupation, sex, education, Age, observed the bite, under influence of alcohol, admission, snake bought to hospital | | | | | | | | | |
| c. Dependent Variable: bitetoadmissiontime_min | | | | | | | | | |

**S1 table:** **Hierarchical regression model summary**
