# Supplementary material for: Time delays in treatment of snakebite patients in rural Sri Lanka and the need for rapid diagnostic tests
Source: PLoS Negl Trop Dis. 2020 Nov 30;14(11):e0008914. doi: 10.1371/journal.pntd.0008914 (PMC7728389; doi:10.1371/journal.pntd.0008914)
Supplement: S2 Table — (DOCX) [file pntd.0008914.s002.docx]

**S2 table: Variables in the hierarchical regression model contributing to the bite to admission time (dependent variable)**

| **Coefficients^a^** | | | | | | | | | | | | |  |  |
| --- | --- | --- | --- | --- | --- | --- | --- | --- | --- | --- | --- | --- | --- | --- |
| Model | | Unstandardized Coefficients | | Standardized Coefficients | t | Sig. | 95.0% Confidence Interval for B | | Correlations | | | Collinearity Statistics | | |
|  |  | B | Std. Error | Beta |  |  | Lower Bound | Upper Bound | Zero-order | Partial | Part | Tolerance | | VIF |
| 1 | (Constant) | 131.62 | 59.08 |  | 2.23 | 0.03 | 15.62 | 247.62 |  |  |  |  | |  |
|  | Age | 0.55 | 0.71 | 0.03 | 0.78 | 0.44 | -0.84 | 1.94 | 0.04 | 0.03 | 0.03 | 0.86 | | 1.17 |
|  | sex | -22.09 | 21.59 | -0.04 | -1.02 | 0.31 | -64.47 | 20.30 | -0.04 | -0.04 | -0.04 | 0.96 | | 1.04 |
|  | education | -15.37 | 13.97 | -0.05 | -1.10 | 0.27 | -42.80 | 12.06 | -0.04 | -0.04 | -0.04 | 0.89 | | 1.13 |
|  | occupation | 0.77 | 3.34 | 0.01 | 0.23 | 0.82 | -5.79 | 7.33 | -0.01 | 0.01 | 0.01 | 0.79 | | 1.27 |
| 2 | (Constant) | 291.64 | 77.58 |  | 3.76 | 0.00 | 139.30 | 443.97 |  |  |  |  | |  |
|  | Age | 0.43 | 0.70 | 0.03 | 0.61 | 0.54 | -0.95 | 1.81 | 0.04 | 0.02 | 0.02 | 0.84 | | 1.19 |
|  | sex | -27.91 | 21.66 | -0.05 | -1.29 | 0.20 | -70.44 | 14.61 | -0.04 | -0.05 | -0.05 | 0.93 | | 1.08 |
|  | education | -18.87 | 13.85 | -0.06 | -1.36 | 0.17 | -46.06 | 8.32 | -0.04 | -0.05 | -0.05 | 0.88 | | 1.14 |
|  | occupation | -0.72 | 3.39 | -0.01 | -0.21 | 0.83 | -7.38 | 5.93 | -0.01 | -0.01 | -0.01 | 0.74 | | 1.35 |
|  | under influence of alcohol | 0.63 | 42.27 | 0.00 | 0.02 | 0.99 | -82.37 | 83.64 | 0.01 | 0.00 | 0.00 | 0.97 | | 1.03 |
|  | observed the bite | -73.32 | 20.87 | -0.14 | -3.51 | 0.00 | -114.29 | -32.34 | -0.15 | -0.14 | -0.13 | 0.93 | | 1.08 |
|  | snake bought to hospital | -34.44 | 24.17 | -0.06 | -1.43 | 0.16 | -81.90 | 13.03 | -0.08 | -0.06 | -0.05 | 0.91 | | 1.10 |
|  | admission | -53.33 | 23.39 | -0.09 | -2.28 | 0.02 | -99.25 | -7.40 | -0.08 | -0.09 | -0.09 | 0.93 | | 1.08 |
| a. Dependent Variable: bite to admission time_(min) | | | | | | | | | | | | | | |
|  | | | | | | | | | | | | |  |  |
