# Supplementary material for: Time delays in treatment of snakebite patients in rural Sri Lanka and the need for rapid diagnostic tests
Source: PLoS Negl Trop Dis. 2020 Nov 30;14(11):e0008914. doi: 10.1371/journal.pntd.0008914 (PMC7728389; doi:10.1371/journal.pntd.0008914)
Supplement: S1 Data — (PDF) [file pntd.0008914.s003.pdf]

Anuradhapura Snakebite Cohort - data collection sheet

Study ID of the pt: *(Tick "✓ " the appropriate space only if the data present)*

A – Demographic

1. Patient's Name : 2. BHT: 3.Age: 4.Sex:
5. Address: 13. Weight:: kg 14. Height: m

6. Occupation:
7.GS Division: 8.Div. Secretary:
9.Contact no (if any): (a) , (b) , (c)
10. Highest educational level: < O/L ( ) O/L ( ) A/L ( ) HE ( )
11. Involvement in farming: Full time ( ) Part time ( ) No inv ( )
12. Number of dependents:

B – Snake bite

1. Date: 2. Time: AM/PM 3. Place:
4. Activity: 5. Alcohol influence: ( )
6. Transport to primary care: Mode: 7. Distance: km
8. Snake seen : yes ( ) no ( ) 9. Delay in finding transport: mins
10. Intentional delays for treatment seeking: yes ( ) no ( ) 11.duration: mins
12. Reasons: a. Native treatment: ( ) b. Waited for symptoms: ( )
c. snake capturing ( ) d. Waited for someone to accompany to hospital ( )

C – Bite & snake

1. Patient sure of the snakebite : Yes ( ) No ( )
Write the complaint in his own words (sinhala)

3. Fang marks: 1 ( ) 2 ( ) 3 ( ) 4 ( ) several marks in U shape ( )
4. Provoked bite ( )
5: Snake was seen while bite occur: ( ) 6: ID by patient:
7: Specimen brought to hospital ( ) 8. Available on admission to THA ( )
9. Snake ID by MO/ CRA: 10. Snake ID by AS:
11. EIA diagnosis:

2. Site of bite: *(draw, indicating fang marks)*

D – Pre-admission

1. Tourniquet : Yes ( ) No ( ) 2. Pressure bandage: Yes ( ) No ( )
3. Time since bite when applied: min 4. Width: cm 5. Applied duration: min
6. $Ca(OH)_2$: ( ) 7. Incision: ( ) 8. Lemon: ( ) 9. Smashed onion: ( )
10: Oral sucking : ( ) 11. Washing of bite site ( ) 12. Soap: ( ) 13: Splinting ( )
14: Paracetamol ( ) 15. Other pain relievers (specify) :
16: Native/ Ayurveda treatment : Yes ( ) No: ( ) 17: herbal decoctions (nasna): ( )
18. Snake stone ( ) 19. Other interventions of native/ Ayurveda :

E – Hospitals

1. Admission type: Direct: ( ) Transferred ( )
2. Primary care: a. DOA: b. TOA: am/pm c. Reason for Tx:
3. Secondary care: a. DOA: b. TOA: am/pm c. Reason for Tx:
4.TH Anuradhapura a. DOA: b. TOA: am/pm

Samples

1. Blood samples: Admission ( ), T1 ( ), T4 ( ), T8 ( ), T 12( ), T24 ( ), T 48 ( )
T72 ( ), T96 ( ), T120 ( ) 2. sfEMG

1. Diabetes Mellitus ( )	2. CKD ( )	3. Hypertension ( )	4. Alcoholic liver disease ( )	**F**
4. Snakebite ( )	a. species:	b. Year:	c: Hospitalized days:	**Past Med**
	a. species:	b. Year:	c: Hospitalized days:	
5. Agrochemical poisoning ( )	6. Seizure disorders ( )	7. Other (specify)		

H: Investigations

	Dt	tm	Result	Dt	tm	Result	Dt	tm	Result	Dt	tm	Result
1. RBC												
2. WBC												
3. Platelets												
4. Hb												
5. WBCT20												
6. CT (lab)												
7. PT												
8. INR												
9. APTT												
10. U RBC												
11. U Prtn												
12. Sr Crtn												
13. BUN												
14. SGPT												
15. SGOT												
16. pH												
17. Na^+												
18. K^+												
19. CPK												

19. ECG/USS abd/Blood picture

G a.: Clinical features (non-neurological)							
**Date**							
**Time point**	Admission	T12	T24	T48	T72	T96	T120
**Time**							
Pulse rate							
BP							
Bleeding from bite site +/-							
Local pain +/-							
Swelling (if +*ve*, state the extension)							
Regional lymphadenopathy +/-							
Blistering +/-							
Bluish discoloration +/-							
Local tissue necrosis +/-							
Gangrene +/-							
Secondary Infections of bite site +/-							
Headache +/-							
Nausia +/-							
Vomiting +/-							
Abdominal pain (indicate grading)							
Diarrhea +/-							
Spontaneous gum / nasal bleeding +/-							
Bleeding from bite wounds +/-							
Bleeding from IV site +/-							
Haematemesis +/-							
Malena +/-							
Haematuria +/-							
Oliguria +/-							
Loin pain +/-							
Anuria +/-							
Black colored urine +/-							
Jaundice +/-							
Tender liver +/-							

I. AVS								
Cycle	No of vials	nfusion rate	Time started	Time finished	Brand and Bt/N	Pre med (*indicate the dose*)		
1					B / V	Adr	HCZ	CPM
2					B / V	Adr	HCZ	CPM
3					B / V	Adr	HCZ	CPM
4					B / V	Adr	HCZ	CPM

J: Adverse reactions								
	**Cycle**	**Brown grading***	**Time of appearance**	**Time of disappearance**	**Adrenaline**	**IV HCZ**	**IV CPM**	**Other (specify)**
1	1							
2	2							
3	3							
4	4							

***Brown grading:**

Grade 1 (mild: **skin and subcutaneous tissue only**) : Generalized erythema, urticaria, periorbital oedema or angioedema

Grade 2 (moderate: features suggestive of **respiratory, CVS or GI involvement**): Dyspnoea, stridor, wheeze, nausea, vomiting, dizziness, diaphores chest or throat tightness, abdominal pain.

Grade 3 (Severe: hypoxia, hypotension or neurologic compromise): Cyanosis or SpO_2 =<92% any stage, SBP <90mmHg, confusion, collapse, LOC, incontinence.

K. Other Management						
**Empirical Antibiotics**						
	**Day 1**	**Day 2**	**Day 3**	**Day 4**	**Day 5**	**Day 6**
**IV crystalloids**						
**Oral**						
**Cryoprecipitate**						
**FFP**						

**Intubation**	Date	Time	Indications:
**Extubation**	Date	Time	
Complications with Ventilation:			

**Dialysis**		HD	PD	**Number of cycles**		Complications:
**ICU care**		Duration d				

**Fasciotomy**		**Wound toilet**	
**Wound infections**			
**Amputations**			

1. Patient Status (outcome): Discharged alive/Death/transferred /LAMA/Missing
2. Status Date & time
3. If the patient dies, Cause of death according to post mortem
4. If patient transferred, Reason for transfer and hospital:

Data collected by: Date:

Review date

L

Status

	Date & time						
	Time point from bite (T)	**Onset time**	**Resol. time**	**TA**	**T1**	**T4**	**T8**
**1.**	**Neuro-muscular paralysis**						
1.1.	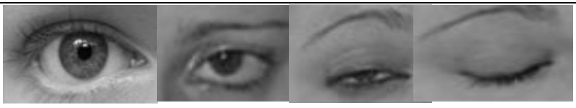 Ptosis0/I/II/III						
1.2.	Diplopia with primary gaze						
1.3.	Diplopia with up-down gaze (+/-)						
1.4.	Diplopia with medial-lateral gaze (+/-)						
1.5.	Eye movements (mark X for directions to which eye movements are weak)						
1.6.	Blurred vision +/-						
1.7.	Pupil reaction (N-normal / D-fixed dilated)						
1.8.	Bulbar weakness (Cough reflex) +/-						
1.9.	Ability to swallow saliva +/-						
1.10.	Inability to talk (dysarthria) +/-						
1.11.	Voice changes (**H**igh pitch/**L**ow pitch /**A**bsent)						
1.12.	Palatal weakness (raising of the arches)+/-						
1.13.	Neck flexion grade (1-5)						
1.14.	Neck extension grade (1-5)						
1.15.	Upper limb tone (**N**ormal/**H**igh/**L**ow)						
1.16.	Lower limb tone (**N**ormal/**H**igh/**L**ow)						
1.17.	Biceps power (1-5)						
1.18.	Quadriceps power (1-5)						
1.19.	Biceps jerk (**E**xaggerated/**D**iminished/**N**ormal)						
1.20.	Knee jerk (**E**xaggerated/**D**iminished/**N**ormal)						
1.21.	Peak Expiratory Flow rate						
1.22.	Tidal volume						
	**Time point**	**Onset**	**Resol.**	**TA**	**T1**	**T4**	**T8**
2.	**Autonomic features**						
2.1.	Excessive sweating +/-						
2.2.	Tearing +/-						
2.3.	Chemosis +/-						
2.4.	Paralytic ileus (auscultate for bowel sounds) +/-						
2.5.	Dairrhoea +/-						
2.6.	Pulse rate						
2.7.	BP (lying)						
2.8.	BP (Sitting)						
2.9.	Urinary incontinence +/-						
2.10.	Fecal incontinence +/-						
**3.**	**Miscellaneous**						
3.1	Paraesthesia / Numbness in the site of bite+/-						
3.2	Reduced hearing (acc. Pt.) +/-						

3.3 Facial muscle fasciculations +/-						
**4. Myotoxicity**						
4.1 Muscle pain in bitten limb +/-						
4.2 Tender muscles in bitten limb +/-						
4.3 Generalized muscle pain +/-						
4.4 Generalized muscle tenderness +/-						
**5. Central effects**						
3.1 GCS						
3.2 Unilateral weakness (L/R)						
3.3 Quadriparesis						

Muscle power grading: No muscle contraction is visible – **0**; Muscle contraction is visible but there is no movement of the joint-**1**; Active joint movement is possible with gravity eliminated – **3**; The muscle group can overcome gravity and move against some resistance from the examiner -**4** ; Full and normal power against resistance - **5**.
